# Observational, longitudinal study of delirium in consecutive unselected acute medical admissions: age-specific rates and associated factors, mortality and re-admission

**DOI:** 10.1136/bmjopen-2015-007808

**Published:** 2015-11-13

**Authors:** ST Pendlebury, NG Lovett, SC Smith, N Dutta, C Bendon, A Lloyd-Lavery, Z Mehta, PM Rothwell

**Affiliations:** 1 Oxford NIHR Biomedical Research Centre, John Radcliffe Hospital, Oxford, UK; 2 Departments of General (Internal) Medicine and Geratology, John Radcliffe Hospital, Oxford, UK; 3 Stroke Prevention Research Unit, Nuffield Department of Clinical Neurosciences, John Radcliffe Hospital and the University of Oxford, Oxford, UK

**Keywords:** INTERNAL MEDICINE, Delirium, Mortality, Readmission, Outcome

## Abstract

**Objectives:**

We aimed to determine age-specific rates of delirium and associated factors in acute medicine, and the impact of delirium on mortality and re-admission on long-term follow-up.

**Design:**

Observational study. Consecutive patients over two 8-week periods (2010, 2012) were screened for delirium on admission, using the confusion assessment method (CAM), and reviewed daily thereafter. Delirium diagnosis was made using the Diagnostic and Statistical Manual Fourth Edition (DSM IV) criteria. For patients aged ≥65 years, potentially important covariables identified in previous studies were collected with follow-up for death and re-admission until January 2014.

**Participants:**

503 consecutive patients (age median=72, range 16–99 years, 236 (48%) male).

**Setting:**

Acute general medicine.

**Results:**

Delirium occurred in 101/503 (20%) (71 on admission, 30 during admission, 17 both), with risk increasing from 3% (6/195) at <65 years to 14% (10/74) for 65–74 years and 36% (85/234) at ≥75 years (p<0.0001). Among 308 patients aged >65 years, after adjustment for age, delirium was associated with previous falls (OR=2.47, 95% CI 1.45 to 4.22, p=0.001), prior dementia (2.08, 1.10 to 3.93, p=0.024), dependency (2.58, 1.48 to 4.48, p=0.001), low cognitive score (5.00, 2.50 to 9.99, p<0.0001), dehydration (3.53, 1.91 to 6.53, p<0.0001), severe illness (1.98, 1.17 to 3.38, p=0.011), pressure sore risk (5.56, 2.60 to 11.88, p<0.0001) and infection (4.88, 2.85 to 8.36, p<0.0001). Patients with delirium were more likely to fall (OR=4.55, 1.47 to 14.05, p=0.008), be incontinent of urine (3.76, 2.15 to 6.58, p<0.0001) or faeces (3.49, 1.81–6.73, p=0.0002) and be catheterised (5.08, 2.44 to 10.54, p<0.0001); and delirium was associated with stay >7 days (2.82, 1.68 to 4.75, p<0.0001), death (4.56, 1.71 to 12.17, p=0.003) and an increase in dependency among survivors (2.56, 1.37 to 4.76, p=0.003) with excess mortality still evident at 2-year follow-up. Patients with delirium had fewer re-admissions within 30-days (OR=0.32, 95% CI 0.09 to 1.1, p=0.07) and in total (median, IQR total re-admissions=0, 0–1 vs 1, 0–2, p=0.01).

**Conclusions:**

Delirium affected a fifth of acute medical admissions and a third of those aged ≥75 years, and was associated with increased mortality, institutionalisation and dependency, but not with increased risk of re-admission on follow-up.

**Strengths and limitations of this study**

- Large unselected (inclusive) cohort with long-term follow-up.
- All patients screened for delirium on arrival and daily thereafter.
- Delirium diagnosis made by the physician/geriatricians admitting and managing the patient.
- No interobserver test of reproducibility of delirium diagnosis.
- Covariables not collected for patients aged <65 years old.

## Introduction

Delirium is an acute and fluctuating confusional state usually associated with an underlying medical disorder.^1–3^ Although delirium is prevalent, and associated with increased care needs and poor outcomes, there is significant uncertainty as to actual delirium rates and associated factors within the UK hospital system,^1^ ^2^ and, elsewhere, there are relatively few studies of unselected cohorts containing more than a few dozen subjects, particularly with longer-term follow-up.^2^ ^3^ Accurate age-specific estimates of delirium rates are necessary to inform service development, particularly in light of increasing numbers of frail elderly people, and recent evidence of poor care in some hospitals.^4–6^

Previous studies have shown that mortality is increased during and up to 3 years after admission with comorbid delirium, but most are from selected samples or from data collected outside the past 10 years, or do not correct for confounders.^1–3^ There is also uncertainty around the impact of delirium on risk of re-admission. Recent studies have highlighted the increased risk of emergency re-admission in the immediate postdischarge period, particularly among patients aged >75 years,^7^ ^8^ but the impact of delirium status during the index admission is unclear. One study from Chile found that delirium did not increase re-admission rates despite the fact that risk factors for delirium and for re-admission might be expected to be similar.^9^

We therefore determined, in a consecutive cohort of patients admitted to our acute medicine team, the age-specific rates of delirium; and then, for patients aged >65 years, we determined the factors associated with delirium, and its impact on mortality and re-admission on long-term follow-up of 2 years.

## Methods

### Patient cohort

The Oxford University Hospitals Trust (OUHT) provides services for all acute medicine patients in a population of approximately 500 000, and runs an unselected medical admissions system, with the majority of patients remaining under the admitting team. In a prospective observational audit, including all consecutive admissions (no exclusion criteria) to a single team over two 8-week periods (September–November 2010 and April–June 2012), patients were screened for delirium on arrival and daily thereafter by the STP/SCS admitting team until discharge, transfer or death. The audit was undertaken to inform future service development, and was approved by the Divisional Management and registered with the OUHT Audit Team (audit registration (datix) number 2197). All data were routinely acquired as part of standard patient care.

## Delirium ascertainment

All patients were seen within 24 h of admission by an experienced consultant physician (dually accredited in acute general (internal) medicine and geriatrics (STP, SCS)) responsible for the patient's care and at least every other day thereafter until discharge, transfer or death. Delirium rates were determined for the cohort overall with risk factor data focused on those aged ≥65 years, since it was anticipated that delirium rates would be low in younger patients.^1^ ^3^ All patients aged ≥65 years or those aged <65 years with confusion or altered behaviour had the confusion assessment method (CAM) examination^10^ and a cognitive test: cohort 1 (2010) had the Mini-Mental State Examination (MMSE)^11^ and cohort 2 (2012) had the abbreviated mental test score (AMTS),^12^ since this was more feasible to perform in the acute medicine setting. The cognitive test and CAM formed part of the clerking pro forma (see online supplementary appendix figure 1) administered by junior doctors on the STP/SCS admitting team, all of whom were trained in their use as part of standard OUHT practice led by STP. Patients aged <65 years did not receive routine admission cognitive testing or CAM from junior staff, and were screened using the CAM by STP/SCS on the postadmission ward round. Cognitive impairment was defined as AMTS <9 or MMSE <24 according to published cut-offs and/or prior diagnosis of dementia.^13^ ^14^ Delirium diagnosis was made according to Diagnostic and Statistical Manual Fourth Edition (DSM IV) criteria^15^ by the responsible physician (STP, SCS) after discussion with the rest of the medical team and was categorised as any delirium (occurring at any point during admission), prevalent delirium (on admission or within the first 48 h) or incident delirium (occurring after the first 48 h). If delirium was present on admission, a 48 h period without evidence of delirium was required before a new episode of delirium occurring during admission could be recorded.

Demographic data, presenting complaint and potential associates of delirium available from routine patient assessment were recorded from the patient, relatives and primary care physician (general practitioner (GP)) and medical records including living arrangements (care home vs home with care package vs home without formal care), number of comorbidities and clinical and physiological parameters (see below). Prior diagnosis of dementia was recorded if the diagnosis was present in the GP letter, reported by the patient or relative or had been recorded previously in the patient's notes. The Charlson index for comorbidities was calculated for all patients.^16^ Physiological parameters on admission (pulse, temperature, systolic and diastolic blood pressure, and respiratory rate) were taken from the patient's chart. Systemic inflammatory response syndrome (SIRS) was used as a measure of illness severity since it required only routinely collected clinical data and was classed as positive if two or more of the following were present: heart rate >90 bpm, temperature <36°C or >38°C, respiratory rate >20 breaths per minute, white cell count <4×10^9^ or >12×10^9 ^cells/L.^17^

The malnutrition universal screening tool (MUST, at risk=>1)^18^ and Pressure Sore Prediction Score (PSPS, at risk=>6)^19^ for pressure area vulnerability were routinely recorded by nursing staff. Urinary or faecal incontinence, falls, constipation requiring intervention (new laxative prescription or bowel care) and sleep deprivation were documented prospectively. Length of stay was calculated for the time spent in the acute hospital. Increase in care needs at discharge was defined as new placement, or new or increased level of care package at home, or discharge to community hospital for rehabilitation. Follow-ups for deaths until 1 January 2014 were performed using electronic hospital records.

### Statistical analyses

Baseline characteristics of patients with delirium were compared to those without delirium using t test and Mann-Whitney U test, as appropriate, for continuous variables and χ^2^ for categorical variables. Potential factors associated with delirium were selected based on national guidelines^2^ and a recent review.^3^ ORs were calculated for univariable associations between potential factors and delirium, unadjusted as well as adjusted for age. In view of the number of potentially important covariables identified in previous studies, we highlighted those variables significant at p=0.001, namely, those variables that remained significant after adopting a Bonferroni correction. Outcomes assessed were chosen on the basis of previous literature reports and included length of stay >7 days, increased care needs on discharge (new or increased package of care or new care home), discharge to care home and death (during admission and on follow-up), and were adjusted for potential confounders including illness severity, premorbid dependency and prior dementia. Emergency re-admission rates on follow-up within the first 30 days and thereafter were determined for the whole cohort and by delirium status, without adjustment for other factors. To determine the independent associates of delirium, preadmission and during-admission univariable associates of delirium significant at the p<0.05 level were entered into two separate multivariable logistic regression models with forward selection. Items included in the SIRS score were not entered separately into the models. Prior to modelling, variables were assessed for collinearity (tolerance statistic <0.4), and all had tolerances of >0.5. The significant risk factors from each model were then entered in a further multivariate logistic regression to obtain the independent associates of delirium using both preadmission and during admission factors.

## Results

**Figure 1:**
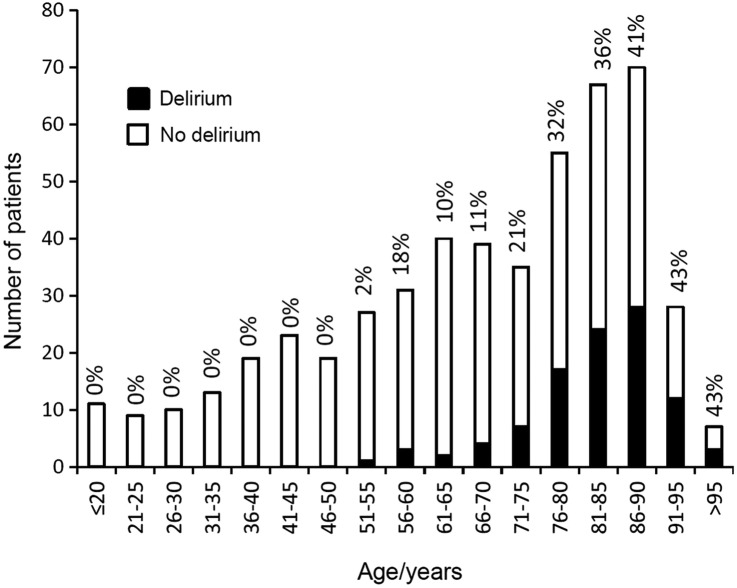
Age-specific rates of delirium in an unselected consecutive cohort of 503 patients admitted to one team in acute general medicine over a 4-month period, showing the proportion with delirium shaded black in each age category.

Five hundred and three consecutive patients (median age 72, range 16–99 years, 236 (48%) male) were admitted over the 4-month period by our acute medicine team. Any delirium occurred in 101 patients (20%; 71 were prevalent, 30 incident and 17 had recurrent episodes). Delirium was rare in younger patients but common in those over 75 years: 6/195 (3%) for <65 years versus 10/74 (14%) for 65–74 years and 85/234 (36%) for ≥75 years (figure 1). Of the six patients aged <65 years with delirium, one patient, from a care home, had severe multiple sclerosis and an indwelling catheter, and was admitted with urosepsis (SIRS score=2); one had a history of alcohol excess and schizoaffective disorder (SIRS score=2); one had a fever and background of cardiac disease (SIRS score=2); one had severe LRTI (SIRS score=3); and one had alcohol withdrawal (SIRS score=1).

There were 308 patients aged ≥65 years (mean/SD age 81/8 years, median=82 years, 164 (54%) female) in whom rates of cognitive impairment were similar using MMSE <24 (49/137 (36%)–cohort 1) and AMTS<9 (70/171 (41%)–cohort 2). In those with prevalent delirium, the presenting complaint more often included confusion or altered behaviour (36/67 (54%) vs 5/233 (2%), p<0.0001) with a trend to less chest pain (6/67 (9%) vs 42/233 (18%), p=0.08). Regarding admission characteristics, those with any delirium were older (mean/SD age 84.0/7.1 vs 79.9/8.4 years, p<0.0001) and more likely to have known dementia (26 (27%) vs 25 (12%), p=0.001), but the number of comorbidities was similar (mean/SD 3.9/1.6 vs 4.0/2.3, p=0.73; mean/SD Charlson index 1.9/1.7 vs 1.9/1.8, p=0.62). Patients with any delirium had lower admission cognitive scores (mean/SD AMTS 5.6/2.4 vs 8.2/2.2, p<0.0001 and mean MMSE 19.7 vs 22.1, p=0.02), lower systolic blood pressure (mean/SD 135.6/34.5 vs 145.7/29.6 mm Hg, p=0.016) with a trend to higher heart rate (mean/SD 88.4/27.6 vs 83.3/18.7 bpm, p=0.11) and a higher pressure sore risk (mean/SD PSPS 8.0/5.6 vs 4.0/4.4, p<0.0001) and malnutrition score (mean MUST score 0.62/0.95 vs 0.33/0.84, p=0.04).

Univariate dichotomised factors associated with any delirium are shown in table 1 with as well as without adjustment for age (see online supplementary appendix tables 1 and 2 for incident and prevalent delirium) and with factors significant at the p=0.001 level shown in bold (ie, with the significance level corrected for the number of variables). Predisposing factors significant at the p=0.001 level corrected for age were history of falls (OR=2.47, 1.45 to 4.22), prior dependency (residence in a care home or at home with a formal care package (OR=2.58, 1.48 to 4.48)) and pressure sore risk (PSPS>6, OR=5.56, 2.60 to 11.88). Abnormal clinical or physiological parameters on admission included cognitive score below cut-off (OR=5.00, 2.50 to 9.99) and clinical dehydration (OR=3.53, 1.91 to 6.53). Diagnosis of infection was strongly related to delirium (OR=4.88, 2.85 to 8.36). Multivariable analysis including all the above factors showed that a cognitive score below cut-off (OR=5.51, 2.59 to 11.70; p<0.0001) and infection (OR=6.80, 3.33 to 13.88, p<0.0001) were independently related to delirium.

**Table 1:** Factors associated with any delirium in patients aged ≥65 years (OR and p values shown unadjusted and adjusted for age), bold values are those significant at the p=0.001 level

Risk factor	Delirium N=95	No delirium N=213	OR	p Value	Adjusted OR	Adjusted p value
Demographic factors						
Age >75 years	85	149	3.65 (1.78 to 7.48)	0.0004		
Female sex	50	118	0.89 (0.55 to 1.45)	0.65	0.77 (0.46 to 1.28)	0.31
Medical history						
Dementia	26	25	2.62 (1.42 to 4.85)	0.0021	2.08 (1.10 to 3.93)	0.024
** **Falls	**45**	**47**	**2.89 (1.72** to **4.87)**	**<0.0001**	**2.47 (1.45** to **4.22)**	**0.0009**
TIA/stroke	30	39	1.89 (1.09 to 3.30)	0.025	1.64 (0.93 to 2.90)	0.088
Depression	22	34	1.56 (0.85 to 2.85)	0.15	1.60 (0.86 to 2.97)	0.14
Other psychiatric history	4	14	0.57 (0.18 to 1.79)	0.34	0.67 (0.21 to 2.14)	0.50
Visual/hearing impairment	16	24	1.48 (0.74 to 2.93)	0.27	1.06 (0.52 to 2.18)	0.87
Charlson score >3	12	25	1.00 (0.48 to 2.09)	1.00	0.95 (0.45 to 2.03)	0.90
Medications >3	76	155	1.12 (0.60 to 2.07)	0.73	0.98 (0.52 to 1.85)	0.94
Medications >7	33	79	0.80 (0.48 to 1.34)	0.40	0.74 (0.44 to 1.26)	0.27
Previous dependency						
** **Care home/care package	**43**	**41**	**3.19 (1.88** to **5.42)**	**<0.0001**	**2.58 (1.48** to **4.48)**	**0.0008**
Care home/community Hospital	20	13	3.82 (1.81 to 8.06)	0.0005	2.88 (1.33 to 6.25)	0.0075
Clinical parameters						
Low cognitive score	**56**	**51**	**5.34 (2.73** to **10.47)**	**<0.0001**	**5.00 (2.50** to **9.99)**	**<0.0001**
Clinical dehydration	**32**	**24**	**3.78 (2.07** to **6.92)**	**<0.0001**	**3.53 (1.91** to **6.53)**	**<0.0001**
Low oxygen saturation	43	66	1.72 (1.03 to 2.84)	0.037	1.66 (0.99 to 2.78)	0.055
Abnormal temperature	25	28	2.18 (1.19 to 4.01)	0.012	2.19 (1.17 to 4.09)	0.014
Abnormal WCC	46	61	2.18 (1.32 to 3.62)	0.003	2.06 (1.23 to 3.45)	0.006
Na <135 mm/L	28	56	1.17 (0.69 to 2.00)	0.56	0.99 (0.47 to 2.10)	0.99
CRP >6 mm/L	75	135	2.17 (1.23 to 3.82)	0.008	2.04 (0.91 to 4.53)	0.082
BUN:Cr ratio	28	47	1.48 (0.85 to 2.55)	0.16	1.41 (0.62 to 3.23)	0.42
SIRS >2	39	52	2.17 (1.29 to 3.63)	0.003	1.98 (1.17 to 3.38)	0.011
** **PSPS >6*	**31**	**20**	**6.05 (2.89** to **12.67)**	**<0.0001**	**5.56 (2.60** to **11.88)**	**<0.0001**
MUST >0†	12	11	2.86 (1.09 to 7.46)	0.032	2.39 (0.89 to 6.43)	0.083
Diagnosis						
Infection	**58**	**51**	**4.93 (2.92** to **8.31)**	**<0.0001**	**4.88 (2.85** to **8.36)**	**<0.0001**
Cardiac	9	41	0.43 (0.20 to 0.92)	0.031	0.37 (0.17 to 0.81)	0.013
Stroke	6	8	1.70 (0.57 to 5.03)	0.34	1.94 (0.64 to 5.90)	0.24
Other	**26**	**117**	**0.29 (0.17** to **0.50)**	**<0.0001**	**0.30 (0.17** to **0.51)**	**<0.0001**
During admission						
Urinary incontinence	**44**	**34**	**4.19 (2.42** to **7.26)**	**<0.0001**	**3.76 (2.15** to **6.58)**	**<0.0001**
Faecal incontinence	**28**	**20**	**3.79 (2.00** to **7.19)**	**<0.0001**	**3.49 (1.81** to **6.73)**	**0.0002**
Bedbound	**34**	**22**	**4.51 (2.45** to **8.31)**	**<0.0001**	**4.21 (2.26** to **7.86)**	**<0.0001**
Sleep deprivation	**26**	**19**	**3.64 (1.89** to **7.00)**	**0.0001**	**3.46 (1.78** to **6.74)**	**0.0003**
Constipation	19	26	1.66 (0.86 to 3.18)	0.13	1.40 (0.72 to 2.73)	0.33
Falls	10	5	4.63 (1.53 to 13.95)	0.0065	4.55 (1.47 to 14.05)	0.008
CT brain scanning	21	23	2.19 (1.14 to 4.20)	0.018	2.49 (1.26 to 4.89)	0.008
Urinary catheter insertion	**27**	**13**	**5.67 (2.77** to **11.64)**	**<0.0001**	**5.08 (2.44** to **10.54)**	**<0.0001**
Outcome						
Stay >7 days	**52**	**58**	**3.22 (1.94** to **5.35)**	**<0.0001**	**2.82 (1.68** to **4.75)**	**<0.0001**
New placement	16	14	3.13 (1.45 to 6.77)	0.004	2.95 (1.35 to 6.45)	0.007
Increased care	26	29	2.66 (1.44 to 4.90)	0.002	2.56 (1.37 to 4.76)	0.003
Death during admission	13	7	4.67 (1.80 to 12.11)	0.002	4.56 (1.71 to 12.17)	0.003

*Missing total n=146.

†Missing total n=201.

Abnormal temperature, temperature >38°C or <36°C; abnormal WCC (white cell count), <4×10^9^ or >12×10^9^ cells per litre; Comm Hosp, community hospital; Low cognitive score, AMTS <9 or MMSE <24; low oxygen saturation, <95% on air;.

BUN, blood urea nitrogen; CRP, C reactive protein; MMSE, Mini-Mental State Examination; MUST, Malnutrition Universal Screening Tool; PSPS, Pressure Score Prediction Score; SIRS, systemic inflammatory response syndrome; TIA, transient ischaemic attack.

During admission factors significant at the p=0.001 level corrected for age were urinary and faecal incontinence (OR=3.76, 2.15 to 6.58; OR=3.49, 1.81 to 6.73), being bedbound (OR=4.21, 2.26 to 7.86), urinary catheter insertion (OR=5.08, 2.44 to 10.54) and sleep deprivation (OR=3.46, 1.78 to 6.74; table 1). Multivariable analysis showed urinary incontinence (OR=3.13, 1.67 to 5.88, p<0.0001), length of stay >7 days (OR=2.63, 1.45 to 4.75, p=0.001) and insertion of urinary catheter (OR=5.50, 2.27 to 13.34, p<0.0001) were independent associates. When all factors including preadmission and during admission factors were entered into the model, cognitive score below cut-off (OR=4.36, 1.93 to 9.85; p<0.0001), infection (OR=6.77, 3.13 to 14.68, p<0.0001), length of stay >7 days (OR=2.49, 1.16 to 5.34, p=0.019) and insertion of urinary catheter (OR=6.26, 1.89 to 20.7, p=0.003) remained significant.

Greater risk of adverse outcomes was seen for delirium after adjustment for age: length of stay >7 days (OR=2.82, 1.68 to 4.75, p<0.0001), discharge with increased care needs (OR=2.56, 1.37 to 4.76, p=0.003) or new care home placement (OR=2.95, 1.35 to 6.45, p=0.007) and death during admission (OR=4.56, 1.71 to 12.17, p=0.003; table 1). The odds of poor outcomes remained broadly similar even after adjustment for SIRS, dementia and preadmission dependency: increased care needs (OR=2.45, 1.28 to 4.70, p=0.007), new placement (2.86, 1.24 to 6.63, p=0.010) and death during admission (OR=3.15, 1.11 to 8.90, p=0.003).

**Figure 2:**
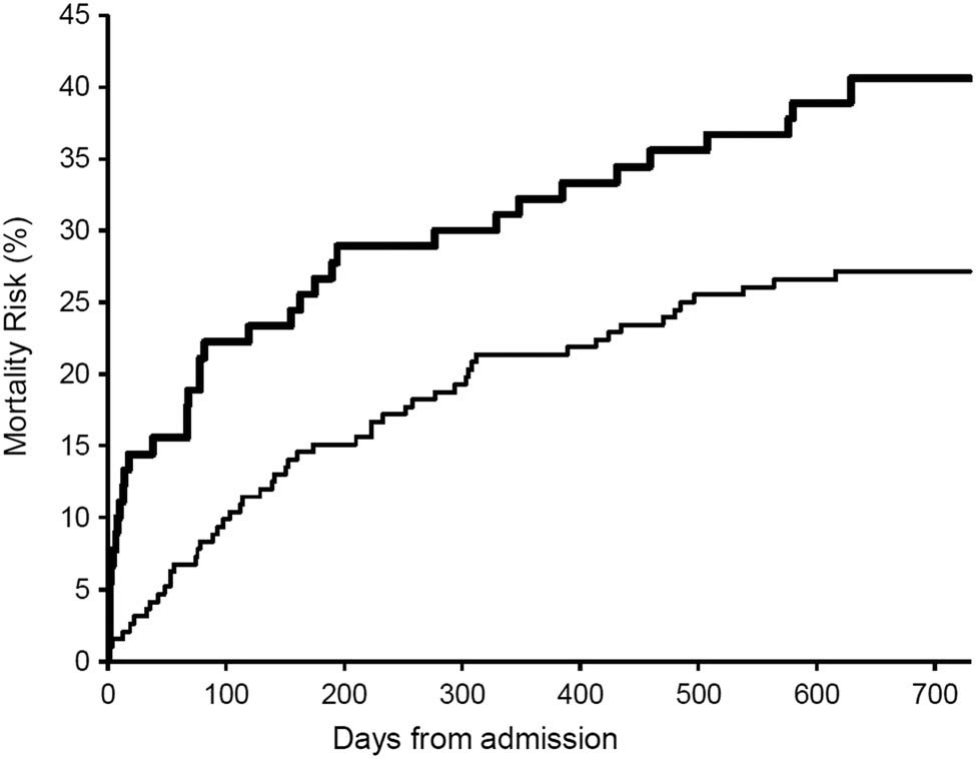
Kaplan-Meier mortality risk curves for consecutive unselected acute general medicine patients aged >65 years with (top line in bold) and without delirium showing high rates of death during admission in the delirium group and similar death rates thereafter up to 2 years’ follow-up (p=0.016).

**Figure 3:**
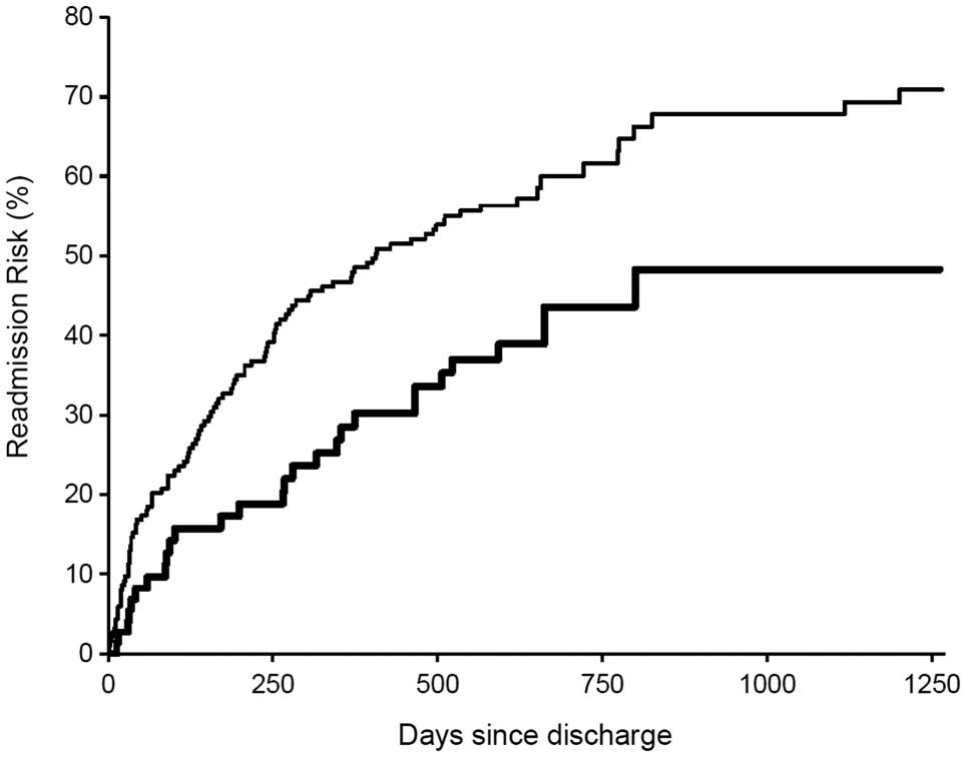
Kaplan-Meier curve for risk of re-admission following discharge for acute general medicine patients aged >65 years with (bottom line in bold) and without delirium during their index admission up to 2 years’ follow-up.

**Figure 4:**
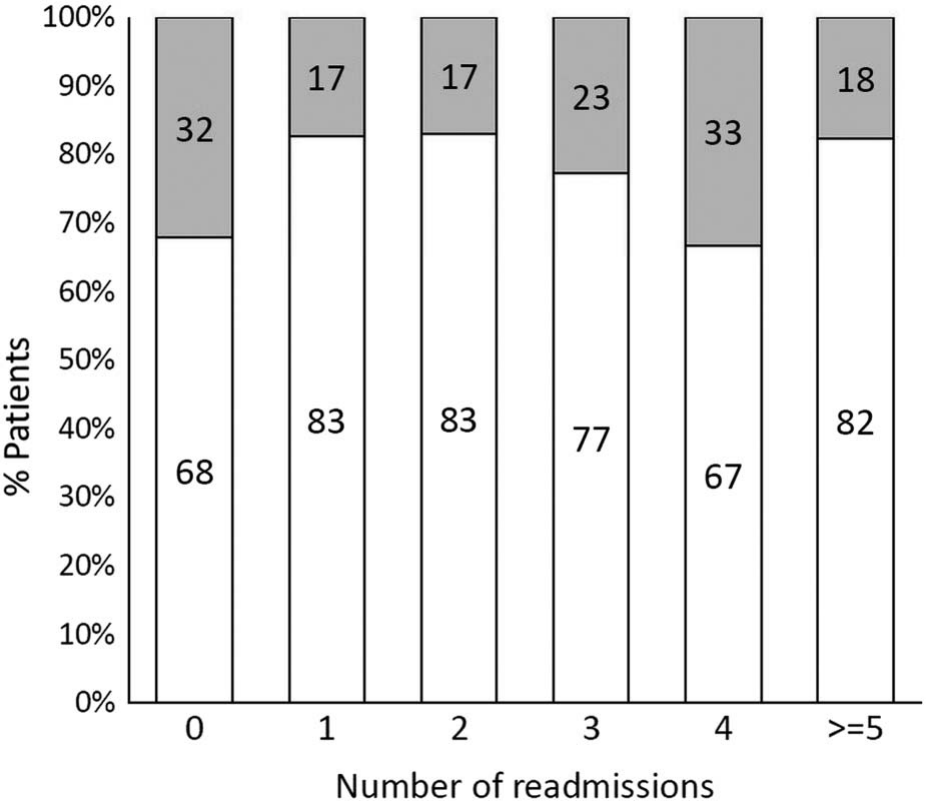
Proportion of acute general medicine patients with 0, 1 or more re-admissions by delirium status at index admission (delirium in grey and no delirium in white, numbers show exact percentages), p trend=0.056.

Mean/SD follow-up time from discharge was 22.4/12.9 months but was non-significantly shorter in patients with delirium (21.3/13.1 vs 22.8/12.8 months). The increased mortality from delirium was maintained throughout the 2 years of follow-up (p=0.016, figure 2) although delirium was not a significant risk for death following discharge after adjustment for confounders. In total, 147 patients were readmitted at least once over the follow-up period and there was an increased risk of admission in the 30 days after discharge: 25 (17%) were admitted within 30 days versus 122 thereafter (OR=24.8, 15.8 to 39.1, p<0.0001). However, patients with delirium at index admission were no more likely than non-delirious patients to be readmitted within 30 days (3/81 vs 22/202, OR=0.32, 0.09 to 1.1, p=0.07) and, in fact, had fewer total re-admissions than non-delirious patients (median, IQR admissions=0, 0–1 vs 1, 0–2, p=0.01 and figures 3 and 4).

## Discussion

Delirium occurred in one-fifth of all adult acute medical in-patients and was more likely to be present on admission than to occur during admission. Only around half of those with delirium on admission had confusion or altered behaviour stated in referral documentation. Delirium was uncommon in those aged <65 years but was over 10 times more likely at age >75 years. Strong associations were seen with predisposing factors including physical and cognitive indicators of frailty, and potentially modifiable factors including dehydration, inflammatory response, infection and catheterisation. The few younger patients with delirium had prior brain insult and or serious illness. Delirium was associated with greater risk of death during admission and with increased care needs on discharge after adjusting for confounders but not with re-admission.

The overall rate of delirium (20%) in our study is consistent with a recent audit in the emergency medicine unit in Braga, Portugal (n=283, mean age 64 years),^20^ and is consistent with recent UK studies restricted to elderly patients; these studies used different methodologies: delirium rate was 37% in Cardiff in consecutive acute medicine admissions (n=273, age >75 years)^21^ and 27% in consecutive emergency acute geriatric, medicine and trauma orthopaedic admissions (aged >70 years) in Nottingham, although frailer patients may have been under-recruited in this study.^22^ Rates are also consistent with reported prevalence of 18–35% and incidence of 11–14% for non-UK general medicine cohorts of at least 100 subjects, which used a validated delirium instrument.^3^

Vulnerability to delirium is related to physical and cognitive frailty, and the related functional dependency.^1^ ^3^ ^20–24^ Our findings suggest that proxy measures of frailty including pressure sore vulnerability, previous dependency and history of falls, may be obtained from routinely collected data, and are useful in pragmatic studies and routine clinical assessment where complex frailty assessment tools are not feasible.^21^ Notably, comorbidity (Charlson index) was not associated with delirium, suggesting that comorbidity cannot be used as a proxy for frailty and that the two represent overlapping but different concepts, as suggested in previous studies.^25^ The high rate of previously unrecognised dementia in hospitalised older people^26^ would explain why low cognitive scores were highly associated with both prevalent and incident delirium, and supports the use of routine cognitive testing in older patients.^24^ There was a trend towards increased risk of delirium with prior history of transient ischaemic attack/stroke, probably because of the strong relationship between cerebrovascular disease and dementia.^27^

Associates of delirium included dehydration, catheterisation, inflammatory response and infection, as supported by other studies,^1–3^ ^20–23^ suggesting that delirium results from the action of inflammatory mediators and possibly changes in cerebral perfusion on a vulnerable brain.^28^ Interestingly, acute cardiac diagnoses showed negative relationships with delirium despite cardiac disease being associated with cognitive decline.^29^ ^30^ Delirium was associated with poor in-hospital outcomes including in-patient falls, incontinence, reduced mobility, longer length of stay and need for increased care on discharge, in keeping with other studies.^1–3^ ^20–22^ Although delirium has been recognised as a risk factor for death in previous studies, many have failed to adjust for confounding factors.^2^ ^3^ We found that delirium remained highly predictive of death during admission over and above the effects of age, illness severity, premorbid dementia and dependency. After discharge, death rates were similar in those with and without delirium to 2 years follow-up.

Surprisingly, delirium at index admission was not associated with increased risk of re-admission. Recent studies have shown an increased risk of re-admission within 30 days of discharge, as seen in our cohort, and it has been proposed that ‘posthospital syndrome’ caused by factors including deconditioning, poor nutrition and sleep deprivation, leads to increased patient vulnerability to new medical problems.^7^ ^8^ Such factors are more prevalent in patients with delirium; thus one might have expected delirium to be associated with increased re-admission risk. It is possible that high rates of death during the index admission leading to healthy survivor effects, increased length of stay in survivors with attention to nutrition, rehabilitation, careful discharge planning and discharge to organisations equipped to care optimally for vulnerable patients might have had protective effects.

Strengths of our study include the prospective inclusive cohort design, continuity of care provided by regular consultant review facilitating delirium diagnosis and examination of factors collected as part of routine clinical care. There are some limitations to our study. First, we did not examine interobserver reproducibility of delirium diagnosis. However, the diagnosis of delirium was made by experienced physicians/geriatricians. Second, since we performed the study in the course of routine care, diagnosis was not blinded to the patients’ clinical characteristics and thus there is the possibility of bias. However, the fact that our observed delirium rate was very similar to rates reported in other studies suggests that there was no significant over-diagnosis. Third, we did not collect risk factor data or outcomes on patients aged <65 years owing to resource constraints, but the numbers of patients with delirium in this group was very small. Finally, a large number of covariables were examined, which may have led to associations occurring by chance. However, covariables were selected based on existing reported associations, and we highlighted those factors that remained significant after correction for the number of variables examined.

In conclusion, our findings have several implications for clinical practice. Rates of delirium are 10-fold higher in the oldest old and fivefold higher in the younger old compared to those aged under 65 years admitted to acute medicine. Delirium is a risk factor for death and increased dependency during admission over and above illness severity, and premorbid functional and cognitive status. Service design and staffing resources should reflect the complex care needs of those with delirium to prevent avoidable deterioration, complications and deaths in this vulnerable group.^3–5^ ^31^ Delirium appears to have less significant effects on mortality over the longer term and does not appear to increase the risk of re-admission within 30 days or thereafter.
